# Bridging Flocculation of a Sterically Stabilized Cationic Latex as a Biosensor for the Detection of Microbial DNA after Amplification via PCR

**DOI:** 10.1021/acs.biomac.3c01187

**Published:** 2024-02-16

**Authors:** Elisabeth Trinh, Lauren J. Batt, Qi Yue, Ruiling Du, Samuel T. Jones, Lee A. Fielding

**Affiliations:** † Department of Materials, School of Natural Sciences, The University of Manchester, Oxford Road, Manchester, M13 9PL, United Kingdom; ‡ Henry Royce Institute, The University of Manchester, Oxford Road, Manchester, M13 9PL, United Kingdom; § School of Chemistry, University of Birmingham, Birmingham, B15 2TT, United Kingdom

## Abstract

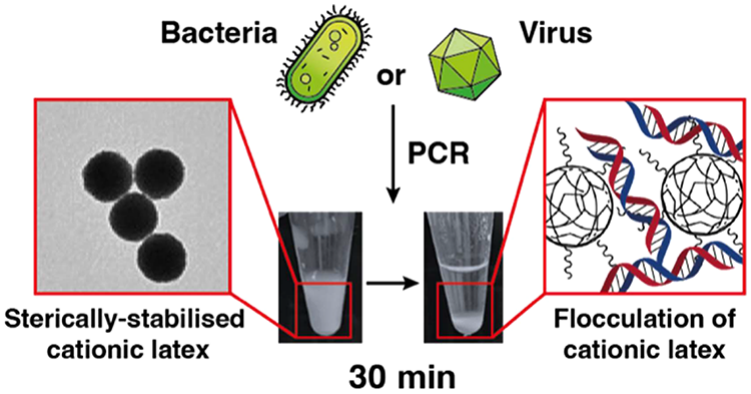

There is a high demand for rapid, sensitive, and accurate detection methods for pathogens. This paper demonstrates a method of detecting the presence of amplified DNA from a range of pathogens associated with serious infections including Gram-negative bacteria, Gram-positive bacteria, and viruses. DNA is amplified using a polymerase chain reaction (PCR) and consequently detected using a sterically stabilized, cationic polymer latex. The DNA induces flocculation of this cationic latex, which consequently leads to rapid sedimentation and a visible change from a milky-white dispersion to one with a transparent supernatant, presenting a clear visible change, indicating the presence of amplified DNA. Specifically, a number of different pathogens were amplified using conventional or qPCR, including *Staphylococcus aureus*, *Escherichia coli*, and Herpes Simplex Virus (HSV-2). This method was demonstrated to detect the presence of bacteria in suspension concentrations greater than 380 CFU mL^–1^ and diagnose the presence of specific genomes through primer selection, as exemplified using methicillin resistant and methicillin susceptible *Staphylococcus aureus*. The versatility of this methodology was further demonstrated by showing that false positive results do not occur when a PCR of fungal DNA from *C. albicans* is conducted using bacterial universal primers.

## Introduction

There is a high demand for point-of-care (POC) assays, which quickly detect genetic material from pathogens and therefore can enable rapid patient diagnosis and effective further treatment.^1,2^ By detecting pathogens accurately, specifically, and quickly, POC assays are a vital tool preventing the spread of infectious disease.^2^ Gram-positive bacteria, including *Staphylococcus aureus*, are a leading cause of nosocomial infections and can lead to potentially life-threatening infections, including endocarditis and sepsis.^3^ Therefore, the rapid and accurate detection of *S. aureus* is important for the diagnosis and consequent antimicrobial therapy. Amplified *Escherichia coli* DNA from a polymerase chain reaction (PCR) has previously been the target of rapid detection methods and biosensors due to the prevalence of food safety related *E. coli* 0157:H7 infections, requiring a rapid and sensitive detection method to prevent the spread of these foodborne illnesses.^4,5^ Herpes Simplex Virus type 2 (HSV-2) is a member of the human Herpesviridae family, affecting approximately 22% of adults.^6^ Patients with HSV-2 infection commonly present with genital lesions, however, HSV-2 can cause life-threatening central nervous system infections such as Herpes Simplex encephalitis and meningitis.^7^ Thus, there is a clear need for POC testing for a wide range of pathogens.

Traditional approaches to pathogen detection include culture, nucleic acid amplification tests, and immunoassay. In traditional bacterial culture methods, the pathogen would be identified using microscopy and Gram staining, and culture would be used to obtain a pure isolate.^8^ However, culture-based methods can take a long time for an accurate diagnosis, which can prolong the period required in order to diagnose a patient, and therefore delays the identification of an appropriate treatment.^9,10^ Viruses can be detected using methods such as the enzyme-linked immunosorbent assay (ELISA) to recognize viral antigens. ELISA and other antigen detection methods have been widely used for the detection of viruses, but reagents can be easily degraded and they can have poor sensitivity.^11^ Lateral flow assays are POC tests and are widely used due to their low cost, ease of use, and rapid results.^12^ However, they have poor sensitivity and specificity in comparison to traditional laboratory testing based on methods such as ELISA and PCR.^13^ Other recommended tests for virus detection include virus isolation, fluorescent antibody tests, and both real time and conventional PCR.^11,14^ PCR and other nucleic acid amplification tests (NAATs) rely on the amplification of nucleic acids, such as DNA or RNA.^15−17^ The benefits of NAATs include their specificity, sensitivity, and that they allow the use of nonpurified clinical samples and the amplification of emerging resistance related genes or mutations.^18^ For a PCR reaction to proceed, the presence of a length of DNA specific to the primers used must be present within the clinical sample, and hence, one of the advantages of PCR is that selectivity of a diagnostic assay can easily be adjusted by changing the primers used.

In addition to the detection of pathogens, identifying appropriate antimicrobial treatment is also a significant concern. Antimicrobial resistance (AMR) is a significant global problem, with the development of new diagnostics outlined as a priority in the U.K. government’s 5 year action plan for AMR.^19^ Infections due to antimicrobial resistant bacteria such as Methicillin-resistant *Staphylococcus aureus* (MRSA) are a major concern for public health.^20^ This is where species display resistance to certain antibiotics such as β-lactams. One particular gene associated with this is the *mecA* gene.^21^ In addition, there are drug-resistant viral strains such as acyclovir-resistant HSV-2.^22^ Currently, detection of antibiotic resistance in bacteria relies on the use of phenotypical testing, including disk diffusion tests, which rely on the exposure of a bacterial isolate to the antibiotics, and observing the inhibition of growth visually.^23^ Multiplex PCR can also be used to accurately identify AMR profiles of both bacteria and viruses, where there are numerous clinically relevant antimicrobial resistance genes.^9,24,25^ The increasing prominence of NAATs and other molecular pathogen diagnosis techniques for POC diagnosis rely on rapid detection, high sensitivity, and specificity. Once DNA has been amplified, this is generally analyzed by gel electrophoresis, which although is an efficient way of detecting the presence of DNA fragments, can be laborious, requires additional equipment, and may be time-consuming.^26,27^ Although qPCR does not require the use of gel electrophoresis after DNA amplification steps, it requires the use of fluorescent dyes such as SYBR green to quantify the DNA.^28,29^

**Scheme 1:**
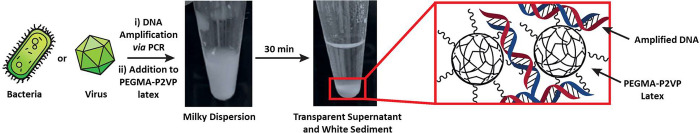
Detection of Amplified DNA *via* Electrostatically Induced Bridging Flocculation of a Cationic Polymer Latex DNA can be extracted directly and amplified *via* conventional PCR. The addition of amplified DNA to a sterically-stabilized PEGMA-P2VP latex causes flocculation and subsequent sedimentation of the milky white latex, providing a rapid and visible method for detecting the success of a PCR. When a PCR is unsuccessful, no amplified DNA will be present and no sedimentation will be observed.

A new approach to detect amplified DNA from conventional PCR using the flocculation of sterically stabilized cationic latexes was recently reported by our group.^30^ Specifically, poly(ethylene glycol methacrylate)-stabilized poly(2-vinylpyridine) (PEGMA-P2VP) latexes flocculated in the presence of negatively charged amplified DNA from *Pseudomonas aeruginosa*, showing visible sedimentation of the latex particles in 30 min without the use of fluorescent labels (Scheme 1). This allowed for the visual detection of amplified DNA without the use of gel electrophoresis, labels, or DNA probes. A key feature of using these latexes is that a clear color change occurs on latex flocculation from a milky-white latex dispersion to an obvious sediment and colorless, transparent supernatant. In addition, the PEGMA-P2VP latex does not flocculate in the presence of other anionic species present in the PCR such as dNTPs. Both of these features would not be the case for, e.g., molecularly dissolved “off-the-shelf” cationic polymers aggregating in the presence of amplified DNA. Thus, the use of this method may reduce the time taken to diagnose and therefore begin appropriate treatment. Herein, the versatility of this approach is significantly extended by demonstrating that it can successfully be utilized to detect common pathogens, including Gram-negative and Gram-positive bacteria (*S. aureus*, MRSA, and *E. coli*) and HSV-2 virus, by changing the PCR primers for these targets. In addition, this flocculation approach is used to detect the antibiotic resistance gene *mecA* in MRSA, and to distinguish between MRSA and methicillin susceptible *S. aureus* (MSSA), which would both require separate treatments if an infection was present.

## Experimental Section

### Materials

2-Vinylpyridine (97%, 2VP; Sigma-Aldrich, U.K.) and divinylbenzene (80 mol % 1,4-divinyl content, DVB; Sigma-Aldrich, U.K.) were passed through a column of activated basic alumina to remove inhibitors and impurities before use. 2,2′-Azodiisobutyramidine dihydrochloride (AIBA; 97%) was purchased from Sigma-Aldrich (U.K.) and used as received. Aliquat 336 surfactant (Thermo Fisher, U.K.) was used as received. Poly(ethylene glycol) methyl ether methacrylate (PEGMA, average *M*_n_ 2000 g mol^–1^, Sigma-Aldrich, U.K.) was supplied as a 50 wt % solution in H_2_O. PCR reagents were used as received. DNA extraction and purification kits (Qiagen, U.K.) were used as per manufacturer’s instructions. qPCR reagents (Thermo Fisher, U.K.) and viral extraction reagents (Invitrogen, U.K.) were used as received.

### Synthesis of PEGMA-Stabilized P2VP Latex via Aqueous Emulsion Polymerization

The preparation of PEGMA-stabilized P2VP latexes *via* aqueous emulsion polymerization has been reported previously.^30,31^ 0.5 g of Aliquat 336 and 1.0 g of PEGMA (*M*_n_ 2000 g mol^–1^) were added to a 100 mL single necked round bottomed flask and stirred at 250 rpm in 38.5 g of deionized water. A comonomer mixture of 2VP (4.95 g) and DVB (0.05 g) was added *via* syringe. The round-bottomed flask was then sealed, and the solution was degassed using five vacuum/nitrogen cycles using a Schlenk line. This was continually stirred at 250 rpm using a magnetic stirrer and then heated to 60 °C in an oil bath. 0.085 g of AIBA was dissolved in 5 g of deionized degassed H_2_O and added to the reaction vessel after 20 min of stirring and heating. The polymerization was allowed to proceed for 12 h at 60 °C, and monomer conversion was determined to be >99% by gravimetry. To remove residual monomers, surfactant, and nongrafted stabilizer, the obtained latexes were purified by dialysis using a membrane (Spectrum Spectra/Por 3 RC Dialysis Membrane Tubing 3500 Da MWCO, Fisher Scientific, U.K.), and 1 L of deionized water which was changed twice daily until the serum surface tension was that of pure water (71 ± 1 mN m^–1^).

### UV–Visible Spectrophotometry

UV–vis absorption spectra were recorded on an Agilent Cary 60 UV–vis spectrophotometer at 600 nm at room temperature. The concentration and purity of the DNA was assessed using a Nanodrop 2000 spectrophotometer (Thermo Scientific Nanodrop ND2000 s/n Q372) or with the use of the Agilent Cary 60 UV spectrophotometer.

### Disc Centrifuge Photosedimentometry (DCP)

Particle size distribution studies were conducted using a Centrifugal Photo Sedimentation (CPS) Disc Centrifuge Model 24000. The calibration standard used was a 348 nm polystyrene latex. Sucrose solution from 12 to 4% w/w in deionized water was used as a density gradient. *n*-Dodecane (0.5 mL) was injected to avoid evaporation, and the spin fluid was allowed to stabilize for 30 min before analysis. Samples were analyzed at disc spin speeds between 20000 and 23000 rpm, and measurements took approximately 30 min for each sample.

### Amplification of Viral DNA Using qPCR

Herpes-Simplex Virus 2 (HSV-2) stocks were initially isolated and verified from clinical samples. Additional virus stocks were grown in Vero cells, isolated, and stored in Dulbecco’s Modified Eagle Medium (DMEM) (Thermo Fisher Scientific, U.K.) supplemented with 1% penicillin/streptomycin (Merck Life Science, U.K.) and 10% heat inactivated fetal calf serum (FCS; Merck Life Science, U.K.). Viral DNA was first extracted using the PureLink Viral RNA/DNA mini kit (Invitrogen) according to manufacturer’s instructions. qPCR was performed with an qPCR instrument (Applied BioSystems StepOnePlus, Thermo Fisher Scientific) and associated software. The following reagents were added to give a total volume of 20 μL. PowerUp SYBR Green Master Mix (2×; Thermo Fisher, U.K.) forward and reverse primers, DNA template, and nuclease free water. The qPCR cycling sequence used was as follows: Stage 1, 95 °C for 10 min; Stage 2, 95 °C for 15 s, followed by 60 °C for 1 min (repeated for 40 cycles); Stage 3, 95 °C for 15 s; Stage 4, 60 °C for 1 min. For each viral sample, the sample analyzed *via* flocculation was compared to both viral cell culture and qPCR analysis, which served as reference assays.

### Amplification of Bacterial DNA Using Conventional PCR

Experiments were conducted using either *Escherichia coli* K12 NCTC 10538, Methicillin-susceptible *Staphylococcus aureus* ATCC 6538, Methicillin-resistant *Staphylococcus aureus* NCTC 11939, or *Candida albicans* as a fungal control. Bacteria and fungi were incubated overnight (12 h) at 37 °C on nutrient agar plates.

If DNA extraction was required, DNA was extracted using QIAamp DNA mini kit (Qiagen) according to manufacturer’s instructions. Otherwise, for colony PCR one colony was used as template DNA for the PCR reaction from a bacterial suspension containing approximately 2 × 10^8^ CFU mL^–1^.

PCR was conducted using 25 μL of Nebnext high fidelity master mix (New England BioLabs, U.S.), 2.5 μL of forward primer (Eurofins Genomics, EU), 2.5 μL of reverse primer (Eurofins Genomics, EU), 1 μL of DNA template from purified target bacteria DNA, and nuclease free water to give a total volume of up to 50 μL. PCR was conducted by using a TGradient PCR instrument (Biometra Göttingen, Germany). The cycle was set as follows: Stage 1, 95 °C for 2 min; Stage 2, 95 °C for 1 min, 53 °C for 30 s, 72 °C for 1 min (repeated for 30 cycles); Stage 3, 72 °C for 5 min.

Following the PCR cycle, to confirm successful PCR, 10 μL of PCR product was mixed with 2 μL of loading dye (Thermo Fisher, U.K.) and analyzed by gel electrophoresis using a 1% w/v agarose gel at 120 V for 90 min. If required, purification of the PCR product was performed using a QIAquick PCR purification Kit (Qiagen). For each bacteria tested, the sample after flocculation was compared to conventional PCR, bacterial culture, and colony PCR, which served as reference assays.

### Statistical Analysis

For the UV–vis spectrophotometry data, a moving average was calculated and normalized using the “Normalize” function on GraphPad Prism 9 (GraphPad Software Inc., CA). Pairwise comparisons between parametric data sets were compared using a student’s *t*-test in GraphPad Prism 9. With additional groups to be compared, statistical analysis was performed using one way analysis of variance (ANOVA) and Tukey’s multiple comparisons test. Differences between groups were considered significant at a *P* value of <0.05.

## Results and Discussion

### Detection of Gram-Negative Bacteria, Gram-Positive Bacteria, and Viruses

Lightly cross-linked, PEGMA-P2VP with a mean diameter of approximately 700 nm was prepared by conventional emulsion polymerization to yield latex particles with a nonionic steric stabilizer and cationic core (Figure SI1). This latex was used in all subsequent studies reported herein.^30^*S. aureus* and *E. coli* were used as Gram-positive and Gram-negative bacteria, respectively, and bacterial DNA was amplified *via* conventional colony PCR after incubation overnight on nutrient agar. The PCR primers used were universal primers that target the 16s gene, meaning the amplicons would be approximately 1400 base pairs (bp) in length (Table 1). HSV-2 DNA was extracted and used as template viral DNA for amplification by qPCR using HSV-2 specific primers, resulting in a PCR product of approximately 80 bp (Table 1). In all cases, amplified DNA was added to latex dispersions and left undisturbed for 30 min. After this time, the success of the PCR was judged by visual observation, whereby a positive result was indicated by sedimentation of the latex (Scheme 1) and a negative result was indicated by the dispersion remaining milky and opaque. Additionally, UV–vis spectroscopy was utilized to monitor the rate of sedimentation on the addition of amplified DNA to latex dispersions.

**Table 1:** Details of the PCR Primers Used

name	target	sequence	amplicon size	specificity
universal bacterial primers^32^	16s rDNA gene	forward primer 27F (5′-AGA GTT TGA TCC TGG CTC AG-3′) and reverse primer 1492R (5′-TAC CTT GTT ACG ACT T-3′)	∼1400 bp	most common bacterial species, not fungi
type III primers^33^	SCC MecIII gene	(5′-CCA TAT TGT GTA CGA TGC G-3′) type III-R (5′-CCT TAG TTG TCG TAA CAG ATC G-3′)	280 bp	methicillin-resistant *Staphylococcus aureus*
HSV-2 primers^34^	DNA polymerase gene	(5′ GAC AGC GAA TTC GAG ATG CTG 3′) reverse (5′ ATG TTG TAC CCG GTC ACG AAC T 3′)	80 bp	Herpes Simplex Virus Type 2

**Figure 1:**
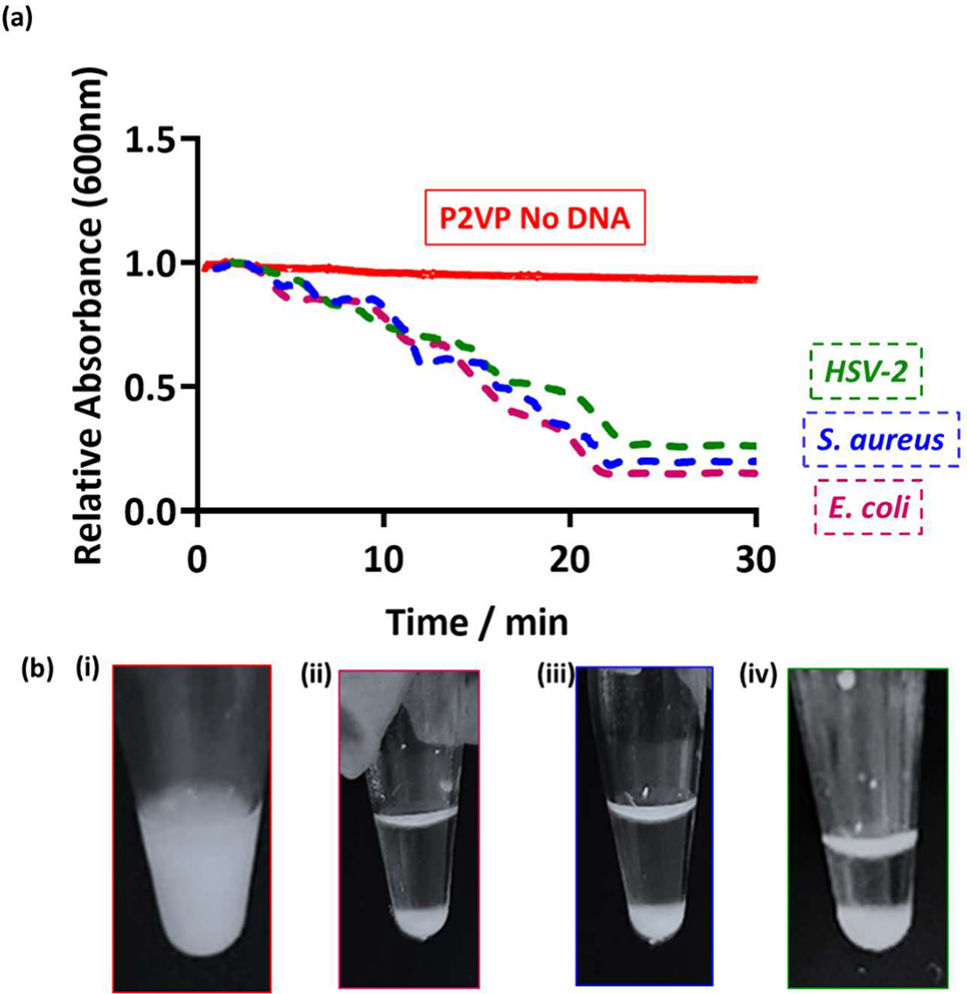
(a) UV–vis spectrophotometry absorbance at 600 nm as a function of time for PEGMA-P2VP latex only (red) and after the addition of DNA to the latex (HSV-2, green; *S. aureus*, blue; *E. coli*, pink). Latex particles were at a concentration of 0.1% w/w and 50 μL of purified amplified PCR product was added. (b) Digital images were taken 30 min after the addition of amplified DNA to 0.2% w/w latex; (i) control with no amplified DNA, (ii) 50 μL of *E. coli* PCR product, (iii) 50 μL of *S. aureus* PCR product, and (iv) 20 μL of HSV-2 PCR product.

It is apparent that sedimentation occurs on the addition of amplified DNA from both Gram-negative (*E. coli*) and Gram-positive (*S. aureus*) bacteria, as well from HSV-2, when added to 0.2% w/w cationic PEGMA-P2VP latex (Figure 1b). In addition, the measured absorbance of 0.1% w/w latex dispersions at 600 nm decreased steadily over ∼20 min after the addition of amplified DNA, reaching almost 0 absorbance after 30 min (Figure 1a). This was expected, as the relatively high molecular weight and negatively charged amplified DNA is capable of electrostatically associating with the cationic latex and causing charge neutralization as well as bridging flocculation. Consequently, when amplified DNA was added to PEGMA-P2VP and monitored by UV–vis, a gradual decrease in the absorbance was observed over 30 min due to latex sedimentation, leaving a transparent supernatant and thus a relatively low absorbance at 600 nm. Additional complementary experiments were performed using disc centrifuge photosedimentometry (DCP) to evaluate the degree of incipient flocculation on addition of amplified DNA to the latex by analyzing the observed particle size distributions. As shown in Figure SI2, shoulders and peaks at larger sizes become apparent in the particle size distributions after the addition of amplified DNA and the subsequent flocculation of the latex particles. These positive results expand upon previous demonstrations from our group showing flocculation in response to amplified DNA from *Pseudomonas aeruginosa*, a Gram-negative bacterium.^30^

Importantly, the PEGMA-P2VP latexes did not show any visual signs of flocculation on the addition of either the DNA extraction reagents or the HSV-2 Mastermix (Figure SI3). Bridging flocculation requires macromolecules (DNA in this case) to be of a certain molecular weight, and the dNTPs, primers and salts present in these mixtures are not of a sufficient molar mass to induce flocculation of the PEGMA-P2VP latex despite having some negative charge or inducing charge screening. Therefore, it can be assumed that the positive results observed herein are due to amplified DNA and not from the reagents used in either extraction or (q)PCR.

### Demonstrating Organism Selectivity

**Figure 2:**
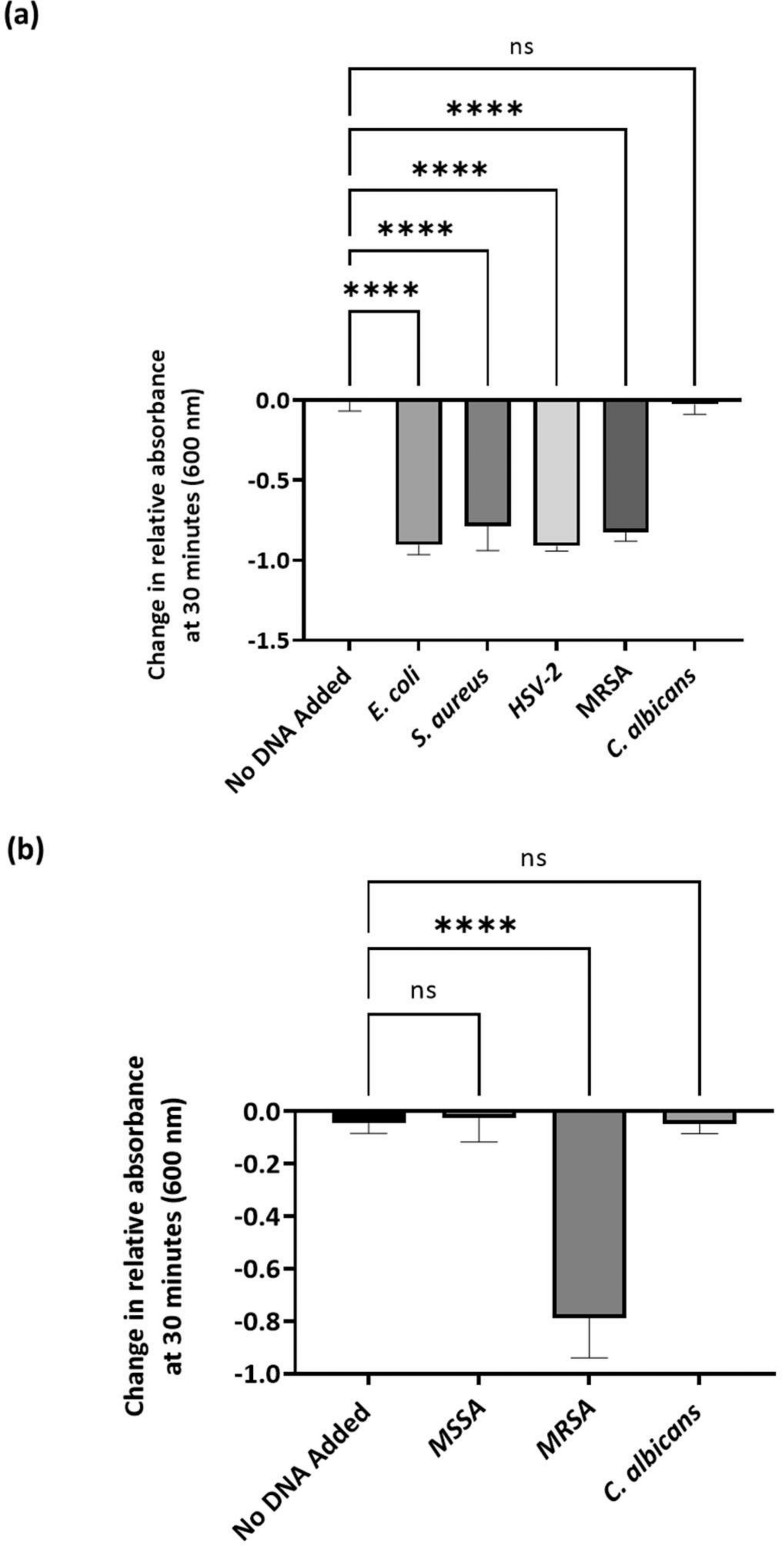
(a) Summary of changes in UV–vis absorbance at 600 nm 30 min after the addition of PCR product from *E. coli*, *S. aureus*, and *C. albicans*, amplified using universal bacterial primers, and HSV-2 amplified using HSV-2 primers to 0.1% w/w PEGMA-P2VP latex. *n* = 3, ****p = <0.01. ns = not significant using ANOVA. (b) Summary of changes in UV–vis absorbance at 600 nm 30 min after the addition of PCR product from MSSA, MRSA, and *C. albicans*, amplified using MRSA-specific type III primers.

As a control experiment, *Candida albicans* was subjected to colony PCR using the universal primers for bacteria previously described (Table 1). PCR product was then added to the latex to observe whether flocculation occurred. As *C. albicans* is a species of fungus and not a bacterium, the PCR should not be successful, and therefore, amplified DNA would not be present in the PCR tube at the end of the colony PCR. After mixing the latex and *C. albicans* PCR product, there was no flocculation or visible sedimentation of the latex, resulting in a milky latex dispersion. Furthermore, no significant change in absorbance was observed *via* UV–vis after 30 min (Figure 2a). All other bacteria previously mentioned (*E. coli*, *S. aureus*, and *Pseudomonas aeruginosa*) showed latex flocculation and sedimentation when amplified by colony PCR with universal primers, demonstrated by a significant change in solution turbidity after 30 min, in comparison to latex only (Figure 2a). Overall, this demonstrates that as long as the primer set used is specific to bacteria (or a given species), it is possible to rapidly distinguish between organisms using this methodology.

### Detection of Antibiotic-Resistant Bacteria

Further experiments were conducted to determine whether the latex would be able to identify the presence of antibiotic-resistant bacteria using methicillin-resistant *Staphylococcus aureus* NCTC 11939 (termed MRSA throughout this study). After incubation overnight on nutrient agar, colony PCR was performed using type III primers (amplicon size of 280 bp; Table 1). As this is a smaller number of base pairs than amplified with the previously used universal primers for bacteria, MRSA was also amplified with universal primers as a control experiment. Methicillin susceptible *S. aureus* (MSSA) was also subjected to colony PCR using type III primers. This species is not resistant to β-lactams, as it does not have the *mecA* gene on the *SCCmec* genetic element. Thus, PCR using these primers would not be successful and amplified DNA would not be present. In addition, the primer set used (type III) did not induce flocculation of the latex (Figure SI4), and therefore, it can be concluded that a successful PCR is required with this specific primer set in order for a positive result to occur.

**Figure 3:**
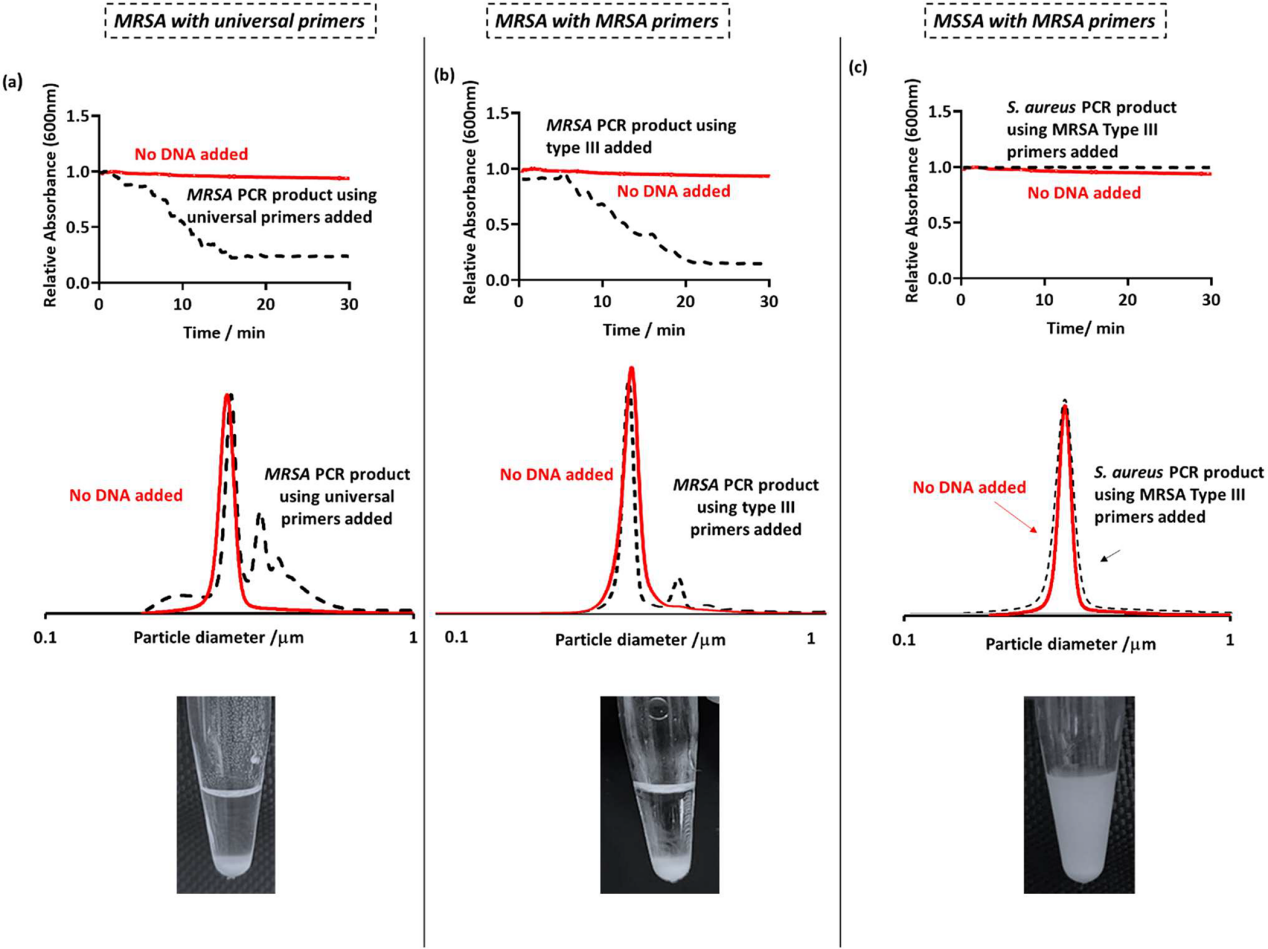
(Top row) UV–vis spectrophotometry absorbance at 600 nm as a function of time for latex only (red) and after the addition of the PCR product to the latex (black). Latex particles were at a concentration of 0.1 w/w % and 50 μL of purified PCR product was added. (Middle row) DCP particle size distributions obtained for PEGMA-P2VP latex (0.01% w/w) on the addition of PCR products from conventional PCR. (Bottom row) Digital images taken 30 min after the addition of 50 μL of PCR product to 0.2% w/w PEGMA-P2VP latex. For the “MRSA primers” columns, MRSA type III primers were used, giving an overall amplicon size of 280 bp. For the left column, universal bacterial primers were used, yielding an amplicon size of approximately 1400 bp.

As previously described, amplified DNA was added to the latex and left undisturbed for 30 min, and further investigations were conducted using DCP and UV–vis to analyze particle size distribution changes and kinetics of sedimentation, respectively. As expected, MRSA was successfully amplified using universal primers, and therefore, the DCP size distributions show flocculation through the appearance of peaks at a higher particle size, shown as the black dotted traces in Figure 3a. This was also demonstrated by visual sedimentation of the latex (Figure 3a) and a statistically significant reduction in UV–vis absorbance over 30 min (Figures 2a and 3b) due to latex sedimentation. MRSA DNA was also successfully amplified using type III primers, as demonstrated by latex flocculation on the addition of PCR product to the latex (Figure 3b). As MSSA does not have a *mecA* gene and is susceptible to β-lactam antibiotics, colony PCR using type III primers was not successful, and amplification of DNA did not occur. Therefore, the results obtained were negative, and the latex dispersion remained milky on addition of the unsuccessful PCR product (Figure 3c). The significant differences in absorbance change over 30 min, in comparison to using these primers with MSSA and *C. albicans* (Figure 2b), indicates that specific genes (in this case MRSA secIII) can be targeted and confidently diagnosed with the aid of this methodology.

### Effect of Source of Bacterial Template DNA

In colony PCR, bacterial cells are heated to a high temperature to release their contents, including the template DNA. Due to the cell contents including proteins and negatively charged components, there was a chance that even before amplification *via* PCR that these components could induce false aggregation and sedimentation of the cationic latex and therefore give a “false positive”. In addition, extraction reagents such as relatively large, negatively charged enzymes may induce unwanted flocculation. These were therefore added to the latex at the concentration used in PCR and assessed *via* UV–vis and DCP in order to see whether flocculation occurred. On addition of the cellular lysate, there was no aggregation of particles, shown by no reduction in absorbance at 600 nm detected by UV–vis (Figure SI5). Overall, this indicates that the flocculation that occurred after amplification using colony PCR was due to successful PCR reactions and the consequent amplification of DNA and not the cellular lysate containing proteins and enzymes present after heating the bacteria.

**Figure 4:**
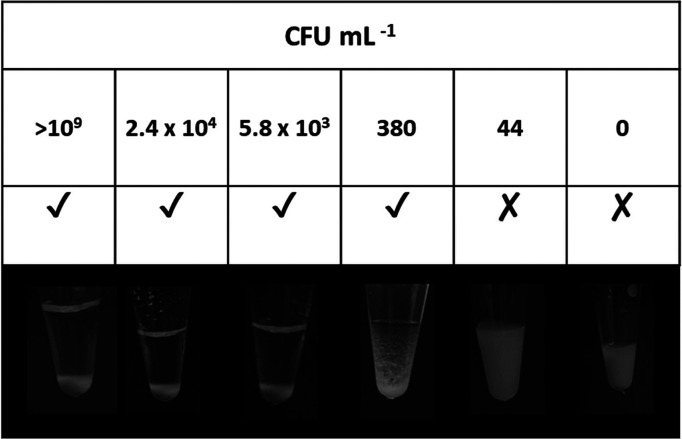
Digital images showing flocculation of PEGMA-P2VP latexes in the presence of amplified DNA from colony PCR, where the bacterial suspension was diluted by a factor of 10 down to 10^6^ and CFU mL^–1^ was measured using colony-counting methods prior to amplification *via* PCR. A tick indicates clear and obvious sedimentation of the latex, a cross indicates that sedimentation did not occur, and a hyphen indicates a borderline result.

When bacteria are incubated overnight, CFU is a microbiological unit that estimates the number of viable bacterial cells and is often used to quantify sensitivity. Laboratory diagnosis *via* culture is often based on colony counts, which reflect the concentration of organisms present in a sample. Hence, to determine the sensitivity of the technique described herein in CFU, bacterial suspensions from an overnight liquid culture (12 h) were diluted by serial dilution of a factor of 10 down to 10^6^, plated on nutrient agar, and CFU mL^–1^ was determined for each dilution by colony counting. These dilutions were used as a template for PCR, with the PCR products then purified and PEGMA-P2VP added to make an overall latex concentration of 0.2% w/w, and particle sedimentation monitored (Figure 4). A clear supernatant was observed for bacterial suspension dilutions of >380 CFU mL^–1^. Even though 44 CFU mL^–1^ was confirmed as positive after colony PCR *via* gel electrophoresis, the DNA concentration when added to the PEGMA-P2VP latex was too low for flocculation to be observed by eye. This establishes a sensitivity limit of this methodology for determining the outcome of direct colony PCR. For reference, many laboratories define a urinary tract infection as the presence of more than 10^5^ CFU mL^–1^ of a single organism,^35^ and many laboratories do not quantify to <10^3^ CFU mL^–1^.^36^ Therefore, the sensitivity of this flocculation-based technique is well below this threshold. In a clinical setting, rapid diagnostic tests are often used alongside other methods that confirm the presence of an infection.^37^ When combined with slower, established methods such as bacterial culture to confirm a true positive result, this gives confidence in a positive result and benefits patients within the clinical setting by providing a quicker time to result for commencement or cessation of antimicrobial treatment. As discussed previously, the response depends on DNA concentration, which can be dependent on a number of factors, including application parameters, amplicon size, and DNA yield.^30^

## Conclusions

This paper demonstrates a method of detecting the presence of amplified DNA from bacterial and viral samples using a sterically stabilized, cationic polymer latex and widely available equipment, providing an accessible alternative DNA detection method. If the targeted DNA is present in a sample, successful PCR results in the obtained amplified DNA inducing flocculation of the cationic polymer latex, followed by rapid sedimentation and visible clear supernatant. If DNA from sources not targeted by the specific primer set used in the PCR was present, this would lead to a negative result, and no flocculation would occur. Specifically, PEGMA-P2VP latex was prepared using aqueous emulsion polymerization and added to amplified DNA using universal primers from 3 common bacterial species, *E. coli*, *S. aureus* and MRSA. The latex was shown to flocculate in the presence of amplified PCR product in cases, as demonstrated by digital images, DCP and UV–vis spectrophotometry. The robustness of this methodology was demonstrated by showing that false positive results do not occur in the presence of bacterial cell lysate, DNA extraction agents, primers in the absence of DNA, or when PCR of fungal DNA from *C. albicans* is conducted using bacterial universal primers. This methodology was demonstrated to be sensitive to bacterial suspension concentrations above 380 CFU mL^–1^, which is below the threshold generally used for determining the presence of an infection. Furthermore, this technique was demonstrated to be able to detect the outcome from qPCR of viral DNA (HSV-2) and diagnose the presence of specific genomes through primer selection (MRSA versus MSSA).

## Supporting Information Available

Additional experimental details; Characterization of PEGMA-P2VP latex particles; DCP analysis on addition of various PCR products to latex; UV–vis absorbance data on addition of primers, bacterial lysate, and DNA extraction reagents to latex (PDF)
