# Supplementary material for: Bridging Flocculation of a Sterically Stabilized Cationic Latex as a Biosensor for the Detection of Microbial DNA after Amplification via PCR
Source: Biomacromolecules. 2024 Feb 16;25(3):1629–36. doi: 10.1021/acs.biomac.3c01187 (PMC10934273; doi:10.1021/acs.biomac.3c01187)
Supplement: Supplementary file 1 — bm3c01187_si_001.pdf [file bm3c01187_si_001.pdf]

Supporting Information

Bridging flocculation of sterically stabilised cationic latex as a biosensor for detection of microbial DNA after amplification *via* PCR

Elisabeth Trinh [a], Lauren J. Batt [a,b], Qi Yue [a,b], Ruiling Du [a,b], Samuel T. Jones [a,b,c], Lee A. Fielding [a,b]*

a. Department of Materials, School of Natural Sciences, The University of Manchester, Oxford Road, Manchester, M13 9PL, UK.
b. Henry Royce Institute, The University of Manchester, Oxford Road, Manchester, M13 9PL, UK.
c. School of Chemistry, University of Birmingham, Birmingham, B15 2TT, UK.
E-mail: lee.fielding@manchester.ac.uk

Additional experimental details

Aqueous electrophoresis and dynamic light scattering (DLS)

Measurements were conducted in the presence of 1 mM KCl using a Malvern Zetasizer Ultra instrument to measure both hydrodynamic diameter and zeta potential. Samples were diluted to 0.1 % w/w and data were averaged over three consecutive runs at 25 °C. Zeta potential was calculated from the electrophoretic mobility using the Smoluchowski relationship. An MPT-3 autotitrator equipped with an auto degasser was used for titration using disposable capillary cells. The solution pH was adjusted by adding either dilute (0.025-0.25 M) HCl or KOH.

Transmission electron microscopy (TEM)

TEM studies were conducted using a FEI Tecnai G2 20 instrument operating at 200 kV and equipped with a Gatan 1k CCD camera. Aqueous polymer dispersions were diluted to approximately 0.1 % w/w using deionised water at ambient temperature. TEM samples were prepared by depositing 2 μl of diluted copolymer dispersion onto carbon-coated copper grids (Agar Scientific, 400 mesh) and dried under ambient conditions for 30 minutes.

Supporting Figures

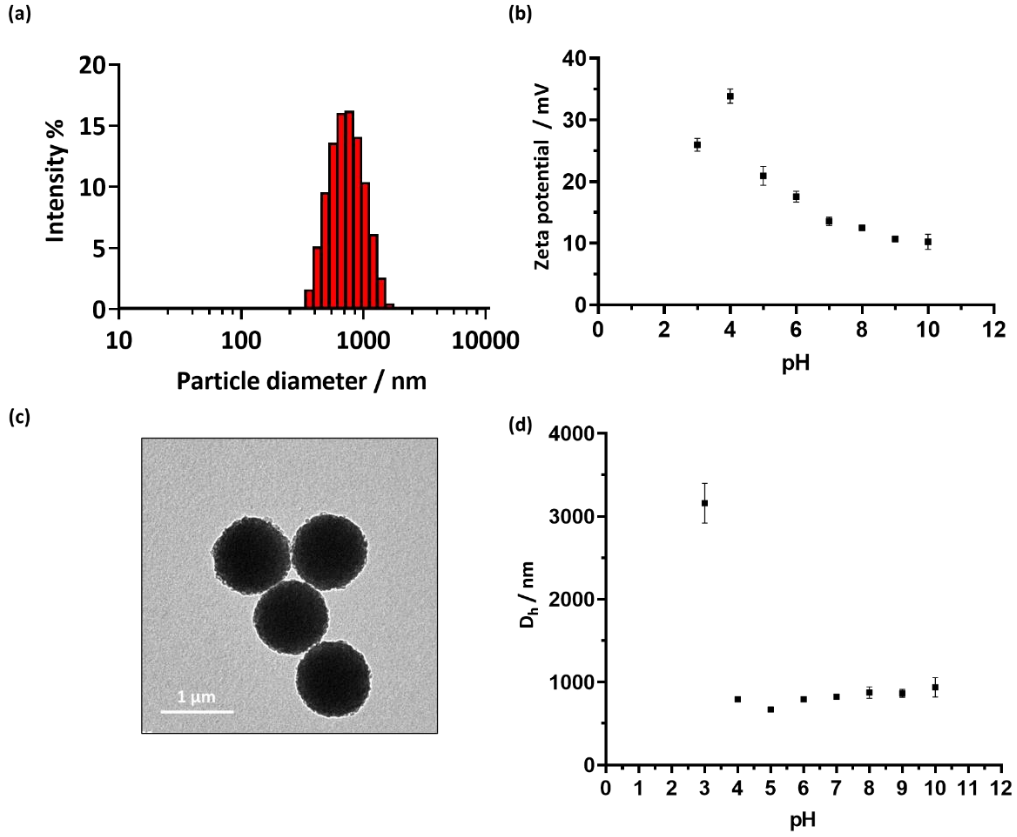

Figure SI1. Characterisation of cationic PEGMA-stabilised P2VP latex particles prepared by aqueous emulsion polymerisation. (a) Particle size distribution measured *via* dynamic light scattering (DLS). (b) Zeta potential as a function of pH. (c) Representative TEM image. (d) Mean hydrodynamic diameter as a function of pH. DLS and Zeta potential measurements were conducted at a latex concentration of approximately 0.1 % w/w with 1 mM KCl as a background electrolyte. The pH was adjusted using KOH and HCl.

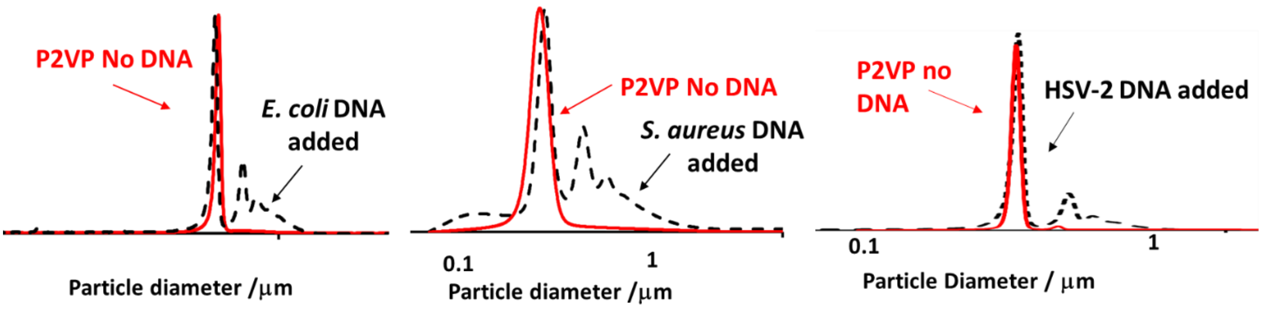

Figure SI2: Disc centrifuge photosedimentometry (DCP) analysis of PEGMA-P2VP latex before and after being added to amplified DNA from PCR for *E. coli*, *S. aureus* and HSV-2. The overall latex concentration was 0.01 %, and the approximate overall DNA concentration was 4 ng µl^{-1}.

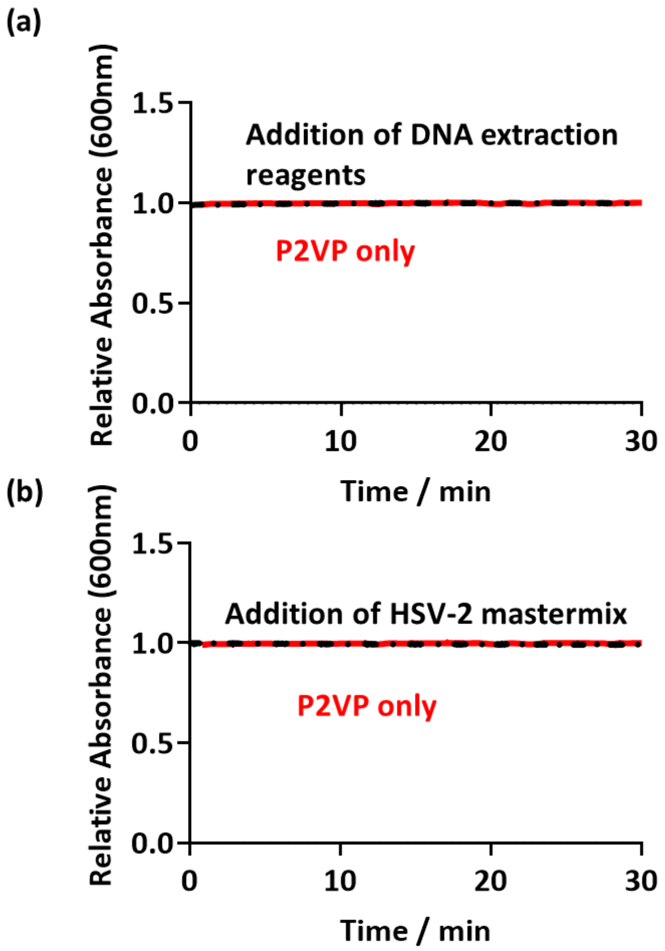

Figure SI3. UV-Vis spectrophotometry absorbance at 600 nm obtained for PEGMA-P2VP latex (red) for; (a) addition of viral DNA extraction reagents; (b) addition of 20 µl HSV-2 PCR mastermix. The latex concentration was 0.1 % w/w and reagents were added at the concentration used during PCR.

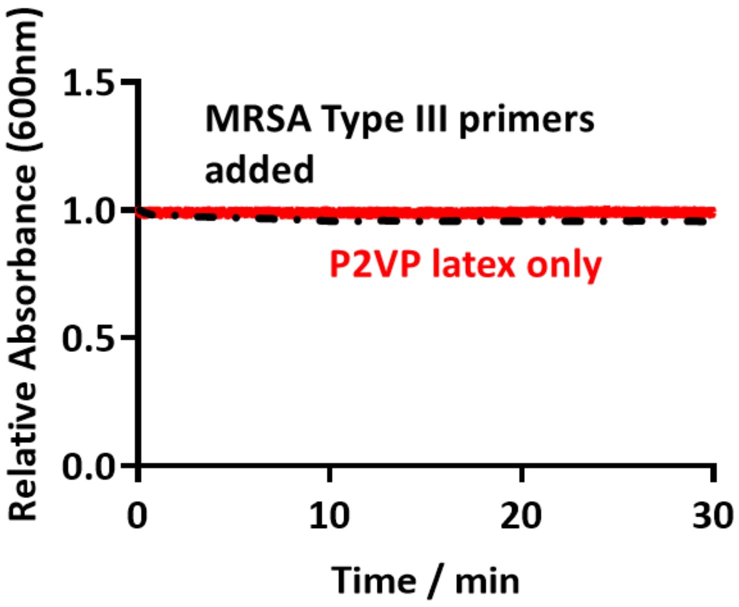

Figure SI4. UV-Vis absorbance spectrophotometry absorbance at 600 nm obtained for PEGMA-P2VP latex (red) and on the addition of MRSA type III primers (black). The latex concentration was 0.1 % w/w, and reagents were added at the concentration used during PCR.

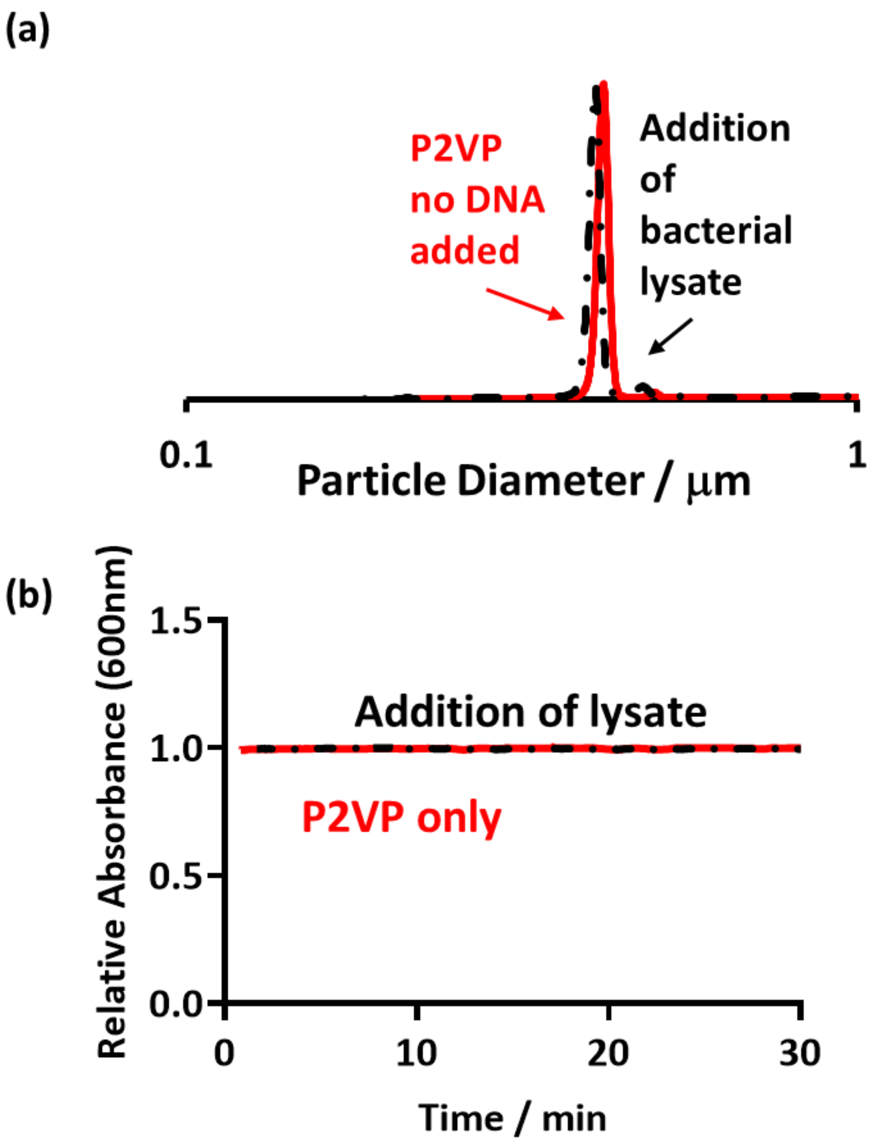

Figure SI5 (a) DCP analysis of PEGMA-P2VP latex before and after being added to bacterial lysate. (b) UV-Vis absorbance spectrophotometry absorbance at 600 nm obtained for PEGMA-P2VP latex (red) and on the addition of bacterial lysate (black). The latex concentration was 0.1 % w/w, and reagents were added at the concentration used during PCR.
